# Biogas potential of organosolv pretreated wheat straw as mono and co-substrate: substrate synergy and microbial dynamics

**DOI:** 10.1038/s41598-024-68904-8

**Published:** 2024-08-08

**Authors:** Omprakash Sarkar, Ulrika Rova, Paul Christakopoulos, Leonidas Matsakas

**Affiliations:** Biochemical Process Engineering, Division of Chemical Engineering, Department of Civil, Environmental, and Natural Resources Engineering, Luleå University of Technology, 971‑87 Luleå, Sweden

**Keywords:** Anaerobic digestion, Bioammonium, Wheat straw, Organosolv pretreatment, Co-fermentation, Bio-fertilizer, Environmental biotechnology, Environmental impact

## Abstract

Anaerobic digestion (AD) technology can potentially address the gap between energy demand and supply playing a crucial role in the production of sustainable energy from utilization of biogenic waste materials as feedstock. The biogas production from anaerobic digestion is primarily influenced by the chemical compositions and biodegradability of the feedstock. Organosolv-steam explosion offers a constructive approach as a promising pretreatment method for the fractionation of lignocellulosic biomasses delivering high cellulose content.This study showed how synergetic co-digestion serves to overcome the challenges of mono-digestion's low efficiency. Particularly, the study evaluated the digestibility of organosolv-steam pretreated wheat straw (WS_OSOL_) in mono as well as co-digesting substrate with cheese whey (CW) and brewery spent grains (BSG). The highest methane yield was attained with co-digestion of WS_OSOL_ + CW (338 mL/gVS) representing an enhanced biogas output of 1–1.15 times greater than its mono digestion. An ammonium production was favored under co-digestion strategy accounting for 921 mg/L from WS_OSOL_ + BSG. Metagenomic study was conducted to determine the predominant bacteria and archaea, as well as its variations in their populations and their functional contributions during the AD process. The Firmicutes have been identified as playing a significant role in the hydrolysis process and the initial stages of AD. An enrichment of the most prevalent archaea genera enriched were *Methanobacterium, Methanothrix*, and *Methanosarsina*. Reactors digesting simpler substrate CW followed the acetoclastic, while digesting more complex substrates like BSG and WS_OSOL_ followed the hydrogenotrophic pathway for biomethane production. To regulate the process for an enhanced AD process to maximize CH_4_, a comprehensive understanding of microbial communities is beneficial.

## Introduction

An interest in bioenergy technology has increased as several countries embrace biogas as a key technology of dealing with a wide variety of organic wastes^1^. Anaerobic digestion (AD) processes have become increasingly significant due to their ability to produce biogas, a renewable form of energy, through the biological treatment of various types of waste and wastewater^2,3^. AD is a highly promising biological system for converting organic waste, including lignocellulosic biomass, food waste, municipal solid waste etc., into energy-rich biogas along with nutrient rich organic fertilizer^2,4^. This conversion process involves a series of stages that rely on the interactions between different groups of bacteria and archaea^5–7^. Therefore, maintaining a delicate balance between these microorganisms and the surrounding environmental conditions is crucial for ensuring the stability and efficiency of AD systems. Waste, particularly biomass waste, represents a significant portion of the materials suitable for anaerobic digestion. The potential of anaerobic digestion to produce biogas from different waste streams depends on factors such as process parameters, environmental conditions, and crucially the nature of substrates^2,5^.

Many studies shown the benefits of co-digestion over mono digestion^8–10^. These benefits include increased digestibility due to synergistic effects caused by co-substrates and greater process stability^11,12^. In co-digestion process, bioCH_4_ output is expected to increase due to the buffering capacity and diverse nutritional profile provided by substrate while the carbohydrate balance the C/N ratio and regulate the ammonia generation. Compared to mono-digestion, biogas output can be significantly increase by 25–400% through co-digestion strategy from the same substrates^13^. The economic viability of AD plants is enhanced by the synergistic interaction between substrates and microorganisms during co-digestion. This is primarily attributed to the increased proportion of bioCH_4_ in the overall biogas yield. The total biogas and bioCH_4_ content are influenced by the chemical compositions and biodegradability of organic sources. For example, the introduction of maize silage (40%, COD) to waste activated sludge resulted in a substantial increase of 154% in CH_4_ compared to single digestion of sludge^14^. According to Zhen et al. (2015), the incorporation of grass into the waste activated sludge (30% w/w) resulted in a significant enhancement in CH_4_ by 12.5% and volatile solids removal by 9.2%^15^.

Ammonia (NH_3_) is often produced during the microbial breakdown of organic feedstock that contains a significant quantity of nitrogenous materials (typically in the form of proteins and urea)^16^. Hydrolysis converts the majority of the Kjehldahl nitrogen to ammonium, therefore cellular production only removes a little amount of nitrogen, whereas about 60–80% of the COD and BOD are transformed into biogas^17^. The majority of the nitrogen in digestate comes from ammonium nitrogen (NH_4_^+^–N), making up between 50 and 80% of the total nitrogen (TN)^18^. Ammonia (NH_3_) plays a crucial role in manufacturing fertilizers, which are essential for ensuring food security globally. NH_3_ has also been recognized as a potential energy source due to its efficient storage and transportation properties, surpassing hydrogen (H_2_). However, the conventional Haber–Bosch (HB) process used to produce NH_3_ demands a considerable amount of energy and leads to greenhouse gas emissions, as it relies on H_2_ derived from fossil fuels. Therefore, it is imperative to develop sustainable strategies for NH_3_ synthesis, especially considering the projected population growth by 2050 and escalating environmental concerns. Prioritizing the use of environmentally friendly raw materials is essential to enhance energy efficiency. Sustainable agriculture is reinforced by a circular economy aimed at maximizing the recovery of essential nutrients. Bio-based nitrogen fertilizers derived from digestate have the potential to replace synthetic mineral nitrogen, facilitating a closed loop of agricultural fertilizer flow^19^. As a result, a bio-based and circular economic system is emerging, focused on improving the recovery of vital nutrients from biological waste streams in a sustainable and environmentally friendly manner, marking a transition from a fossil-based economy^20^.

The aim of this study was to evaluate mono and co-digestion potential of three different substrates (wheat straw, brewery spent grains and cheese whey) towards biogas production. At first, wheat straw (WS) underwent organosolv (OSOLV) pretreatment to efficiently remove hemicellulose/lignin, resulting in a substantial increase in the accessible surface area and a decrease in the degree of polymerization of cellulose. This process greatly enhances the enzymatic digestibility of the straw. The organosolv (OSOLV) pretreatment emerged as a great choice for the biomass fractionation. In the OSOLV process, biomass is pretreated at temperatures ranging from 100 to 220 °C using solvents and acids such as ethanol and sulfuric acid. The final outputs of this process comprise solid materials containing cellulose, an aqueous solution rich in hemicellulose, and a fraction of lignin^21,22^.

Further the study explored the co-digestion of organosolv-pretreated WS solids with nitrogen-rich substrates like brewery spent grains and cheese whey to evaluate their impact on biogas and ammonium production. To provide insight into the synergy of single and co-digestion, we also separately digested untreated WS, BSG, and CW. Combining nitrogen containing substrates with carbohydrate-rich substrates is crucial for maximizing biogas production through anaerobic digestion, leading to enhanced efficiency and stability. Nitrogen (N) and carbon (C) are essential nutrients for the bacteria involved in biogas production. Co-digestion enables the achievement of a balanced carbon-to-nitrogen ratio, which enhances biogas production when compared to digesting individual substrates. It is important to maintain a balanced C:N ratio to support bacterial growth and protein synthesis, resulting in a stronger and more dynamic microbial community. The inclusion of nitrogen from the co-substrate improves the biodegradability of the entire mixture, resulting in increased biogas production. Maintaining a balanced C:N ratio is crucial for ensuring a stable and efficient anaerobic digestion process, which minimizes disruptions and maximizes biogas production in the long run. Furthermore, we delved into studying the microbial dynamics, including bacterial and archaeal communities, to understand the mechanisms and interactions involved in digesting different substrates rich in cellulose, hemicellulose, or proteins. The insights gained from this research could pave the way for the effective bioconversion of lignocellulosic/protein-rich biomass into renewable energy and biobased fertilizers, thus contributing to greener production methods.

## Materials and methods

### Feedstock and inoculum

This study evaluated the anaerobic digestion (AD) potential of three different biomass substrates, including wheat straw (WS), brewery waste grains (BSG), and cheese whey (CW). The BSG and CW were provided by Skellefte Bryggeri (Skellefteå, Sweden), and Norrmejerier, a Swedish company, respectively. Both the BSG and CW are stored at − 20 degrees to maintain their quality until they are utilized. The wheat straw used in this study was cultivated in Denmark. The WS and BSG samples were subjected to an air-drying process and subsequently milled using a knife mill (Retsch SM 300; Retsch GmbH, Haan, Germany) with a 1-mm screen. The resulting milled samples were then stored at room temperature. The biocatalyst used in this study was a mixed culture collected from a biogas plant located in Luleå, Sweden. The sludge was filtered to remove solid particles and settled overnight. The thickened sludge (VS: 0.13 g/g) was used as a biocatalyst after removing the mostly water supernatant. Homogenized BSG analysis revealed 95.1 ± 0.02% w/w total solids, with 92.1 ± 0.02% w/w volatile solids (VS). Cellulose, hemicellulose, and lignin were found to be 28.17%, 15.17%, and 12.28% w/w, respectively.

### Organosolv pretreatment of wheat straw

A hybrid organosolv-steam explosion unit was used for the pretreatment of lignocellulosic wheat straw to extract the cellulosic portion^23^. The resulted solids rich in cellulose from the organosolv pretreatment were used as substrate for AD. Briefly, a total of 200 g of dried WS was mixed with water, acetone, and ethanol in proportions of 40%, 30%, and 30% respectively. Subsequently, an acid catalyst (H_2_SO_4_, 1.1 g) was added to the mixture. After the reactor had been sealed off, the remaining ethanol (needed to get the desired pretreatment ethanol concentration) was pumped into it from outside using an external pump. During the pretreatment phase, water (inside the reactor) was heated to 180 °C using steam (from both within the reactor and an external heating source) for 1 h. Steam was produced in a steam generator located outside of the reactor, and it was transferred into the reactor through pipes. After the pretreatment phase, the discharge valve was opened, resulting in an instant decompression of the reactor. Consequently, the expelled material in the form of slurry was collected through the cyclone apparatus^23,24^. In the next step, using a vacuum filtration, the solid matter was separated. Further, the solid portion was subjected to ethanol wash, followed by air-drying. The liquid portion was processed to extract lignin and hemicellulose, as detailed in previous literature. However, these specific fractions (lignin and hemicellulose) were not included in the scope of the present study^23,24^. Finally, the obtained solids were vacuum filtered from the slurry, washed, and stored at room temperature. The pretreated solids obtained through the hybrid steam explosion technique was used as substrate in this study. The experiments of enzymatic saccharification of pretreated solids were conducted by following the procedure described in our earlier publication^25^. The organosolv pretreated wheat straw was mainly composed of cellulose (0.66 g/g), hemicellulose (0.14 g/g) and lignin (0.17 g/g).

### Anaerobic digestion experiments

**Figure 1:**
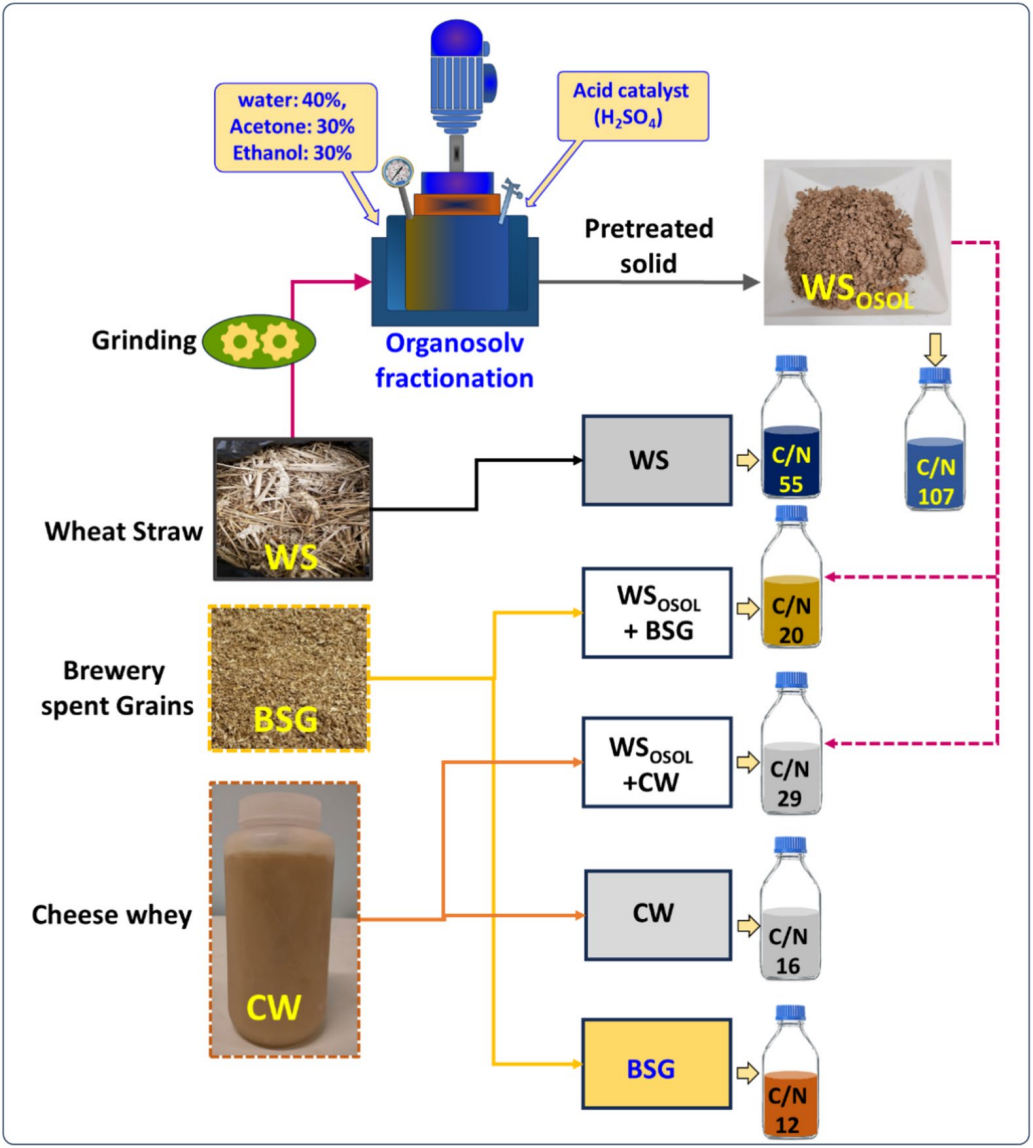
The experimental setup for comparing organosolv pretreated WS (WS_OSOL_) to untreated wheat straw (WS), brewery spent grains (BSG) and cheese whey (CW) in mono and co-digestion strategy.

The AD potential test of all the three substrates (organosolv pretreated wheat straw, brewery spent grains and cheese whey) was evaluated using the Automatic Methane Potential Test System: AMPTS-II from Bioprocess Control AB (Lund, Sweden). Filtered municipal sludge was used as inoculum by considering inoculum-to-substrate volatile solids (VS) ratio of 2. For an effective breakdown of complex lignocellulosic substrates, a higher inoculum-to-substrate ratio was employed thereby enhancing the efficiency of the process. Figure 1 depicts the experimental setup for comparing organosolv pretreated WS (WS_OSOL_) to untreated wheat straw (WS), brewery spent grains (BSG) and cheese whey (CW) in mono and co-digestion strategy. The individual reactor (total volume 1L) of the AMPTS system was specifically designed and fabricated with a glass bottle was connected with a top micro-motor for mixing. The reactors were loaded with a combination of specific ratio of substrate and inoculum, as indicated in Table 1. All the reactors were operated in batch mode at 35 °C for a period of 24 days. Before starting up, the reactors were sparged with nitrogen gas for fifteen minutes to create anaerobic condition. The biogas produced during the AD process was evaluated by connecting the reactors headspace with a gas-flow meter. The volumetric gas production data was recorded utilizing the software that accompanied the reactors. The experiment was performed in triplicate, which involved conducting control digestions in the absence of substrate.

**Table 1:** Mono and co-digestion of organosolv pretreated wheat straw with BSG and CW for biogas and ammonium production.

Reactor	Substrate	VS load (g)	Reactor volume (L)	Temperature (°C)	Anaerobic digestion time (days)	Inoculum-to-substrate ratio	C/N
BSG	Brewery spent grains	10	1	35	24	2	12
CW	Cheese whey	10	1	35	24	2	16
WS_UT_	WS-untreated	10	1	35	24	2	55
WS_OSOL_	WS-organossolv pretreated	10	1	35	24	2	107
WS_OSOL_ + BSG	WS_OSOL_ + BSG	10	1	35	24	2	20
WS_OSOL_ + CW	WS_OSOL_ + CW	10	1	35	24	2	29

### Analytical methods

The total solids (TS) content of the substrates used was determined through the gravimetric method after subjecting them to drying in an oven at a temperature of 110 °C for 24 h. The ash content was determined using a gravimetric method, wherein the samples were subjected to combustion in a muffle furnace at a temperature of 550 °C for 3 h. Volatile solids (VS) was determined by subtracting the ash content from the total solids (TS) content. The quantification of cellulose and hemicellulose in wheat straw samples was performed using the methodology described in our previous publication^23,24^. The change in pH during the AD process were regularly monitored using a pH meter (pHenomenal-pH1100L; VWR, Stockholm, Sweden). Volatile fatty acids (VFAs: acetic, propionic, butyric, and valeric acids) in the digestate was analyzed using high-performance liquid chromatography (HPLC), PerkinElmer system. The HPLC system consisted of a 410 LC pump and a RID-6A refractive index detector (RID). The column Aminex HPX-87H (Bio-Rad in Hercules, CA, USA) with dimensions of 300 × 7.8 mm, was operated at a constant temperature of 65 °C. The mobile phase used was prepared with 5 mM H_2_SO_4_ and was eluted at a flow rate of 0.6 mL/min. The biogas compositional analysis was performed using a mass spectrometer (GAM 400; InProcess Instruments, Bremen, Germany) and an Agilent 990 micro-GC equipped with a COX column (COX UM1MX0.8MMID BF, CP-PORABOND Q, 1MX 0.25 mm × 3Um). The temperature of the column and injector was maintained at 110 °C and 80 °C, respectively. Volatile fatty acid quantification was accomplished with the use of calibration curves based on market available standards (10 mM, volatile free acid mix; Sigma-Aldrich, St. Louis, MO, USA). Ammonium quantification was performed using the Ammonium Test kit (Spectroquant) in conjunction with photometric measurements using the Spectroquant® Move 100. The chemical composition of the biomass was determined by measuring carbon, hydrogen, and nitrogen contents following a standardized procedure on a Euro EA 3000 Elemental Analyzer by flash combustion at 980 °C in tin vials, using 1 to 2 mg of samples (EuroVector, Pavia, Italy).

### Microbial diversity: DNA extraction

On day 24, sludge samples were collected from each of the reactors to evaluate the microbial diversity. The DNA extraction process for the samples was conducted as per the protocol described by FastDNA Spin kit for Soil (MP Biomedicals, USA). Initially in a Lysing Matrix tube, 500 μL of sample, consisting of 480 μL of sodium phosphate buffer and 120 μL of MT buffer was loaded. The bead beating was conducted at a velocity of 6 m/s for 4 × 40 s. The purity and validation of product size of extracted DNA was performed using Tape station 2200 and Genomic DNA screen tapes through gel electrophoresis. Finally, DNA concentration was determined by using the Qubit dsDNA HS/BR Assay kit (Thermo Fisher Scientific, Waltham, MA, USA).

#### Library preparation-DNA sequencing and Bioinformatic processing

The amplicon libraries targeting the variable region 4 (abV4C) of the 16S rRNA gene in bacteria and archaea have been prepared using an Illumina protocol^26^. 10 ng of extracted DNA was used as a template for PCR amplification of the variable region 4 (abV4C) amplicons of the 16S ribosomal RNA (rRNA) gene in bacteria and archaea. The specific procedures for DNA sequencing, library preparation, and bioinformatic processing have been discussed in depth in our previous publication^18^.

## Results and discussion

### Enzymatic saccharification efficiency (ESE)

**Figure 2:**
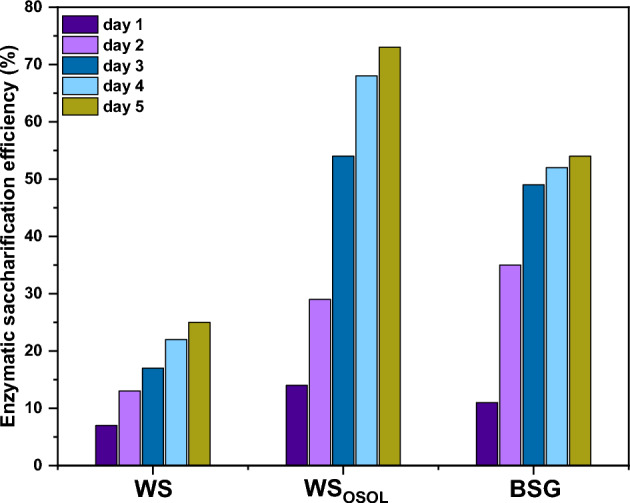
Enzymatic saccharification efficiency of different biomasses (i) untreated, (ii) organosolv pretreated wheat and (iii) brewery spent grains carried out with commercial enzyme cocktail Cellic® CTec2.

The utilization of lignocellulosic biomass is gaining interest as a sustainable alternative for the production of biofuels and energy, aiming to reduce the reliance on fossil fuels and address environmental concerns. In the EU, an annual production of nearly 144 million tons of lignocellulosic wheat straw positions it as a significant agricultural residue in the region. However, the complex chemical composition of wheat straw poses challenges in its effective utilization for biorefinery and bioconversion processes to produce valuable chemicals and fuels. "Organosolv pretreatment offers an effective method for extracting cellulose-rich fractions from wheat straw, which significantly enhances its microbial digestibility and better facilitates its use as a renewable resource. Figure 2 illustrates the enzymatic hydrolysis of organosolv pretreated wheat straw, untreated wheat straw, and brewery spent grains with respect to time. At the end of day 1, the organosolv pretreated wheat straw displayed a higher enzymatic saccharification efficiency (14%) compared to both untreated wheat straw (7%) and brewery spent grains (11%). By day 2, all samples exhibited a notable increase in glucose concentration. The highest enzymatic saccharification efficiency of 35% was observed with brewery spent grains, followed by 29% for organosolv pretreated wheat straw and 13% for untreated wheat straw. The efficiency of saccharification is significantly influenced by biomass composition. Brewery spent grains have lower hemicellulose content compared to wheat straw, which allows for easier access to cellulose for enzyme action. Additionally spent grains generally contain less lignin than wheat straw, which reduces obstacles for enzymes and enhances cellulose accessibility for improved saccharification efficiency. On day 5, the enzymatic saccharification efficiency for organosolv pretreated wheat straw increased by 73%, while brewery spent grains and untreated wheat straw experienced increases of 54% and 25% respectively. Despite the low glucose content at the beginning, organosolv pretreated wheat straw demonstrated easier hydrolysis in the following days, making it an ideal substrate for anaerobic digestion. Conversely, untreated wheat straw exhibited the lowest saccharification efficiency, emphasizing the significance of pretreatment prior to digestion. The organosolv pretreatment process eliminates lignin and breaks down hemicellulose, enhancing the accessibility of cellulose for enzymatic hydrolysis. The cellulose structure enables enzymes utilized in subsequent hydrolysis stages to operate more efficiently, thereby enhancing sugar production. Additionally, the phenolic acids associated with lignin can be significantly decreased through organosolv pretreatment, enhancing fermentation efficiency. These findings also suggest the potential of mixed cultures to catalyze enzymatic hydrolysis and subsequent microbial digestion of readily accessible cellulose^24,27^.

### Biogas production

**Figure 3:**
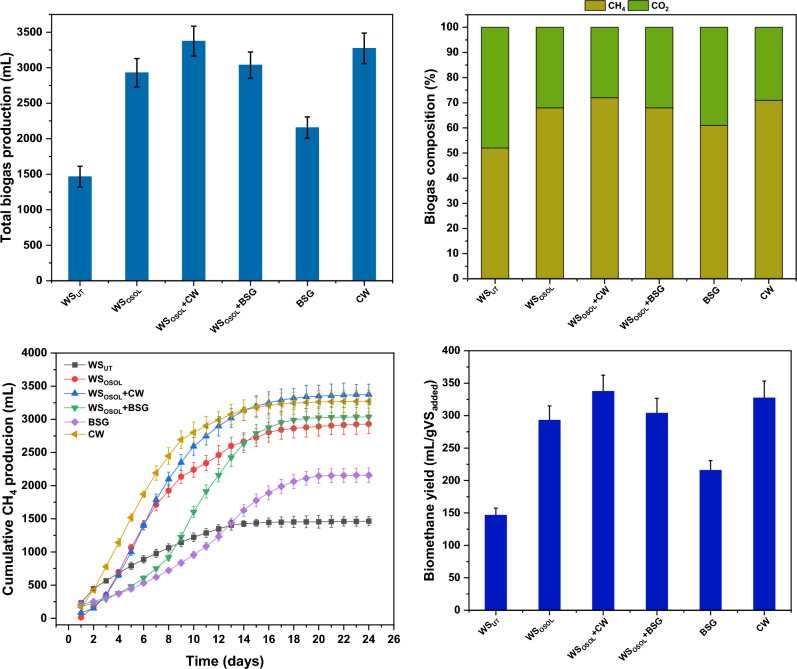
(**a**) Total biogas recorded during mono and co-fermentation of organosolv pretreated wheat straw with brewery spent grains and cheese whey (**b**) biogas composition (**c**) cumulative biomethane evolved (**d**) yields recorded from individual reactor by the end of day 24.

Mono and co-digestion studies were conducted evaluating the compatibility of various selected protein and carbohydrate-rich substrates aiming for an improved biodegradability and microbial metabolites production. Mono and co-digestion of three different substates were investigated for biogas along with ammonium production. The effects of mono and co-digestion on daily biogas production and methane content are shown in Fig. 3. Despite loading of same organic load (VS), the daily biogas production profile from all the reactors significantly varied indicating the nature of substrate. The highest biogas production after 23 days of anaerobic digestion was registered with WS_OSOL_ + CW (4.68 L) followed by CW (4.6L), WS_OSOL_ + BSG (4.46L), WS_OSOL_ (4.3L), BSG (3.53L) and WS_UT_ (2.81 L) representing that CW had been a complimentary to the cellulosic wheat straw in the digestion process. The methane content in the biogas was found to be higher with WS_OSOL_ + CW (72%). On the other side, CW also showed almost similar methane content of 71%, whereas with other rectors, the production was lower than 70% possessing 68% (WS_OSOL_) followed by 68% (WS_OSOL_ + BSG), 61% (BSG) and 52% (WS_UT_). Methane content in the biogas directly influence the volumetric and yield of methane production. The volumetric biomethane production from 10 g VS substrates load resulted with biomethane production of 3375 mL with co-digestion of WS_OSOL_ + CW which was recorded to be the highest followed by CW (3273 mL), WS_OSOL_ + BSG (3037 mL), WS_OSOL_ (2929 mL), BSG (2157 mL) and WS_UT_ (1465 mL). When the biogas profile was examined, it was found that the volumetric biomethane production peaked during the first 5–14 days of operation (> 85%), and afterwards, the production started decreasing. Specifically, within 10 days, more than 50% of the total volumetric methane production occurred, accounting for 85.61%, 76.86%, 76.48%, and 52.78% with CW, WS + CW, WS_OSOL_, and WS_OSOL_ + BSG, respectively, whereas for BSG and WS, the production values were remained at 44.32 and 27.65% respectively. By the end of day 16, almost all the reactors displayed more than 80% of the volumetric production of biomethane except the reactor with untreated WS (78.43%). By the end of day 16, all the reactors showed volumetric biomethane production levels of more than 80% except for the one of the reactors loaded with untreated WS (78.43%) demonstrating the difficulties in the breakdown of lignocellulosic biomass as compared to the same biomass upon pretreatment (WS_OSOL_: 95.77%).

### Methane yields

Co-digestion significantly enhanced the methane yields compared to mono digestion. By the end of the experiment, the highest methane yield was obtained with co-digestion of WS_OSOL_ + CW (337.5 mL/gVS_load_) followed by WS_OSOL_ + BSG (303.7 mL/gVS_load_), whereas among the mono digestion experiments the highest yield was recorded with (CW) 327.3 mL/gVS_load_ which was slightly lower than the co-digestions but higher than other mono digestion setups of BSG (215.7 mL/gVS_load_), WS_OSOL_ (292.9 mL/gVS_load_), and the lowest CH_4_ yield was reported with untreated WS (146.5 mL/gVS_load_). A higher yield here can be connected to the use of readily accessible and easily hydrolysable substrate fractions in CW and organosolv pretreated WS offering variable degrees of degradable components. An appropriate blend of substrate rich and poor in nitrogen with substrate rich in carbon results with C/N ratios that would be regarded as more ideal during co-digestion process^28,29^. Jasko and Dubrovskis (2011) observed a similar trend of biogas production (136–216 mLCH_4_/gVS) from AD of cheese whey^30^. Similar results were also reported from digestion of whey with a methane production of 274 mLCH_4_/gVS^28^. Additionally, these biomasses contain trace elements which are known to be essential to the development of microbes^31^. Most of the elements required for microbial growth and propagation throughout the digestion process are mostly fulfilled by co-digestion.

The observed trend of low methane production, especially in the case of untreated wheat straw digestion, indicates the limited biodegradability of the substrate. The presence of lignocellulosic components in untreated substrates (WS) makes it challenging for the mixed culture to efficiently digest, resulting in slower digestion rates compared to pretreated substrates. Although mixed culture is composed of lignin-degrading and fibrolytic microbes that can relatively digest lignin under anaerobic conditions, the AD demands a suitable pretreatment for an enhanced biogas production^29,32^. Previous studies demonstrated the efficiency of organosolv fractionized soft and hard wood biomass being employed for biogas production representing a yield of 318.6 mLCH_4_/gVS and 176.5 mLCH_4_/gVS from birch and spruce respectively^24^.

The recommended range for the optimal values of C/N for achieving steady performance in anaerobic digestion is 20–60. Previous studies have indicated that C:N ratios ranging from 6 to 9 are considered optimal for the anaerobic digestion of nitrogen-rich waste^33^. Additionally, it was demonstrated that an effective biogas can be produced from anaerobic batch digestion of mixtures of chicken, cow, and piggery waste slurries with carbon to nitrogen ratios of 6:1 and 9:1^34^. In this study, the C:N ratios were determined by calculating the ratio between the carbon and nitrogen content of the substrates. The maximum methane output was obtained by co-digesting CW with organosolv-pretreated WS at a C:N ratio of 29. It is important to note that in all experimental setups, the concentration of ammonia nitrogen during the experiment ranged from 136 to 921 mg/L, significantly lower than the inhibitory level of ionized ammonia of about 1700 mg/L in co-digestion^35^. The increase in methane production can be attributed to the combined effect of nutrients resulting from the synergy of substrates, favorable positive interactions, and a balanced supply of macro- and micro-nutrients.

Despite having a higher C/N value (107) with WS_OSOL_, it still resulted in a higher CH_4_ production compared to BSG and WS, even though their C/N ratios were 12 and 55, respectively. Overall, the co-digestion strategy resulted in greater biogas production compared to mono-digestion. The mono-digestion setup yielded varying levels of biogas production, with CW (C/N: 107.5) generating the highest amount, followed by WS_OSOL_ (C/N: 12.1), BSG (C/N = 12.4), and WS (C/N: 55.4). The high methane yield from cheese whey mono-digestion is due to the presence of easily degradable organic material with a high lactose concentration. Microbes can efficiently break down lactose into simpler compounds, leading to increased CH_4_ production. In this study it was observed that the C/N ratio did not have a significant impact on biogas production. This was because the substrate with a higher C/N ratio was well above the optimum level reported in the literature. Wang et al. (2014) found a noticeable decline in pH levels and methane production once the C/N ratio surpassed 35^36^. In a study conducted by Yan et al. (2015), it was found that maintaining a C/N ratio of 29.6 was crucial^37^. When the ratio fell below this threshold, it resulted in the accumulation of ammonia and subsequently led to high pH levels.

### Effect of mono and co-digestion on ammonium and VFAs production

**Figure 4:**
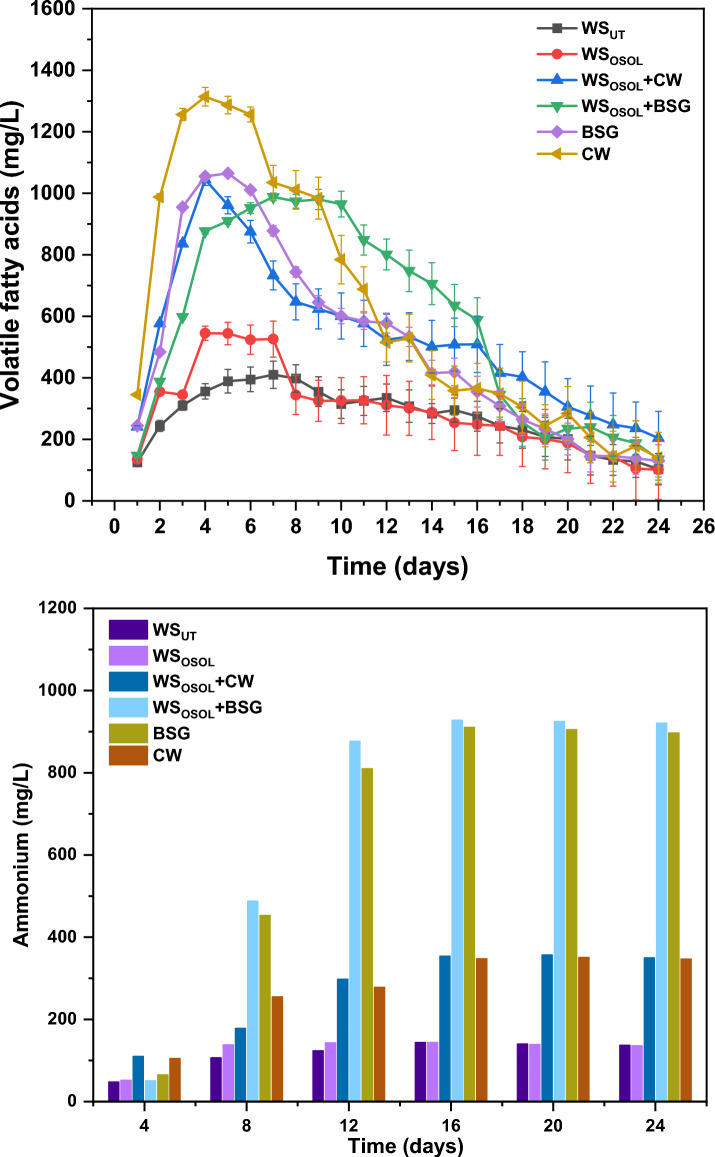
(**a**) Volatile fatty acids and (**b**) ammonium (NH_4_^+^) produced during mono and co-digestion of three different substrate at mesophilic condition.

Ammonia exists in digestate as ammonium ion (NH_4_^+^) and free ammonia (NH_3_)^18^. Decomposition of organic matter during AD, results with methane-rich biogas and digestate rich in and ammonia^38^. The composition of digestate is influenced by the chemical composition of the substrate fed into the reactor. Both mono and co-digestion significantly impact the production of ammonium and volatile fatty acids (VFAs) in the digestate. Ammonium production was consistently observed every 4th day of digestion (Fig. 4). In the initial four days of digestion, ammonium concentration in the digestate ranged from 48 to 110 mg/L. The highest concentration was observed with (WS_OSOL_ + CW) at 110 mg/L, followed by CW at 105 mg/L, BSG at 65 mg/L, WS_OSOL_ at 52 mg/L, WS_OSOL_ + BSG at 51 mg/L, and WS at 48 mg/L. Ammonium accumulation in the digestate gradually increased in all reactors, peaking between days 8 and 16, aligning with biomethane production. Co-digestion of organosolv-pretreated WS and BSG resulted in the highest ammonium production, with concentrations ranging from 488 to 928 mg/L. This can be attributed to the higher fraction of nitrogen (4.2%) compared to CW (2.4%), WS (0.8%) and WS_OSOL_ (0.4%).

The use of nitrogen-rich materials in co-digestion processes offers an exciting potential to increase the production of ammonium (NH_4_^+^). Brewer's Spent Grain (BSG) and cheese whey (CW) are rich in nitrogen, as they contain proteins and other nitrogen-based organic compounds. During anaerobic digestion, these materials undergo microbial processes. The breakdown of proteins leads to the formation of amino acids, which further decompose to release ammonia (NH_3_) and carbon. The balance between ammonia and ammonium, influenced by the pH levels of the digester, plays a crucial role in this system. Co-digestion, involving the simultaneous digestion of multiple types of feedstocks, is a strategy to enhance microbial activity and increase biogas production by combining nitrogen and carbon-rich materials. BSG and its co-digestion show a higher concentration of ammonium due to the presence of 19 amino acids. Even in the case of mono digestion of BSG, a significant production of ammonia ranging from 453 to 911 mg/L has been observed, despite minimal production until day 4. This change in trend is attributed to the cellulosic nature of BSG, which hinders complete degradation by the mixed culture. The gradual breakdown of cellulosic BSG by the mixed culture leads to subsequent ammonium production in later stages alongside biomethane production. BSG, which is composed of hemicelluloses and pectins, is a natural cellulosic material with varying degrees of crystallinity. The microbial enzymatic breakdown of cellulose results in the production of various gases and liquid metabolites, including carbon dioxide, hydrogen, methane, volatile fatty acids, and ammonium. The complexity of cellulosic materials strongly influences their degradation rate. The higher production of ammonium, particularly from BSG, can be attributed to its higher protein content compared to other materials. The yield of ammonia is influenced by the nature of the material in the reactor. The BSG used in this study was found to have a high quantity of protein. Multiple processes, such as proteolysis, peptide degradation, deamination, and deamidation, contribute to the microbial production of ammonium from proteins and their derivatives^18,39,40^. The reactors operated using cheese whey with pretreated wheat straw showed similar levels of ammonium production, ranging from 255–351 mg/L and 178–357 mg/L. In comparison, using only pretreated and untreated wheat straw resulted in the lowest levels of ammonium, with concentrations of 107–144 mg/L. Towards the end of the experiment, the concentration of ammonium decreased in all the reactors, likely due to the microbes using it for growth^40^. There is absolutely no evidence to suggest that ammonia inhibits the AD process. The concentrations of free ammonia nitrogen and ammonium nitrogen should be maintained at around 1700–1800 mg/L, and it is crucial to be aware that N-Kjehldahl becomes toxic at 7 g/L near pH 7.2, effectively preventing the methanogenic process from occurring under mesophilic conditions^41^. Keeping the free ammonia nitrogen and ammonium nitrogen concentrations within specific ranges is important for the anaerobic digestion (AD) process. De Baere et al. (1984) suggested maintaining the free ammonia-N concentration between 80 and 100 for optimal performance^42^. Additionally, Webb and Hawkes observed a 27% decrease in biogas yield when the total ammoniacal nitrogen concentration reached 5980 mg/L^43^.

**Figure 5:**
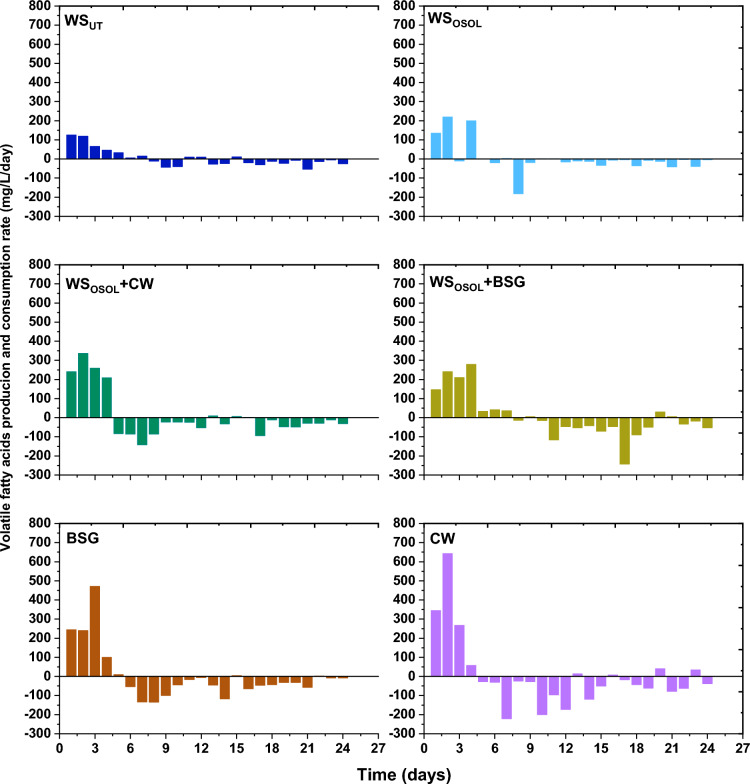
Production and consumption rate of volatile fatty acids in the reactors digesting various substrate.

The simultaneous production and consumption of volatile fatty acids during the process highlights the efficiency of the acidogenic/aceticlastic pathway. These volatile fatty acids were consistently produced and utilized in varying concentrations across all reactors. Notably, the mono fermentation of cheese whey resulted in the highest VFA production of 1315 mg/L, demonstrating its potential as a valuable source. Additionally, the early accumulation of VFAs by acidogens in the reactor signifies the presence of easily accessible reducing sugars, which can assist in activating methanogens for the transformation into methane. The early production of VFA (volatile fatty acids) clearly demonstrated the efficient use of reducing sugars by acidogens and methanogens for methane generation. The determined production (PR) and consumption rates (CR) of VFAs revealed that CW exhibited the highest PR (345–643 mg/L/day), followed by WS_OSOL_ + CW (241–336 mg/L/day), BSG (244–240 mg/L/day), WS_OSOL_ + BSG (147–241 mg/L/day), WS_OSOL_ (135–220 mg/L/day), and WS_UT_ (119–125 mg/L/day) (Fig. 5). VFAs consumption commenced on day 4, with a CR of − 243 mg/L/day observed with WS_OSOL_ + BSG, while other reactors had lower CR rates. It is evident that the production and consumption of VFAs have a direct impact on biomethane yield. Achieving the optimal balance between biomethane and ammonium production is crucial for maximizing yield while simultaneously degrading biomass. The accumulation and utilization of VFAs in the reactor play a significant role in overall digestion process efficiency and metabolite formation. It is important to note that excessive accumulation of VFAs can impede the AD process. This study underscores the significance of achieving a balanced production and consumption of VFAs during mono and co-digestion processes, leading to enhanced biomethane and ammonium recovery.

### Microbial diversity

**Figure 6:**
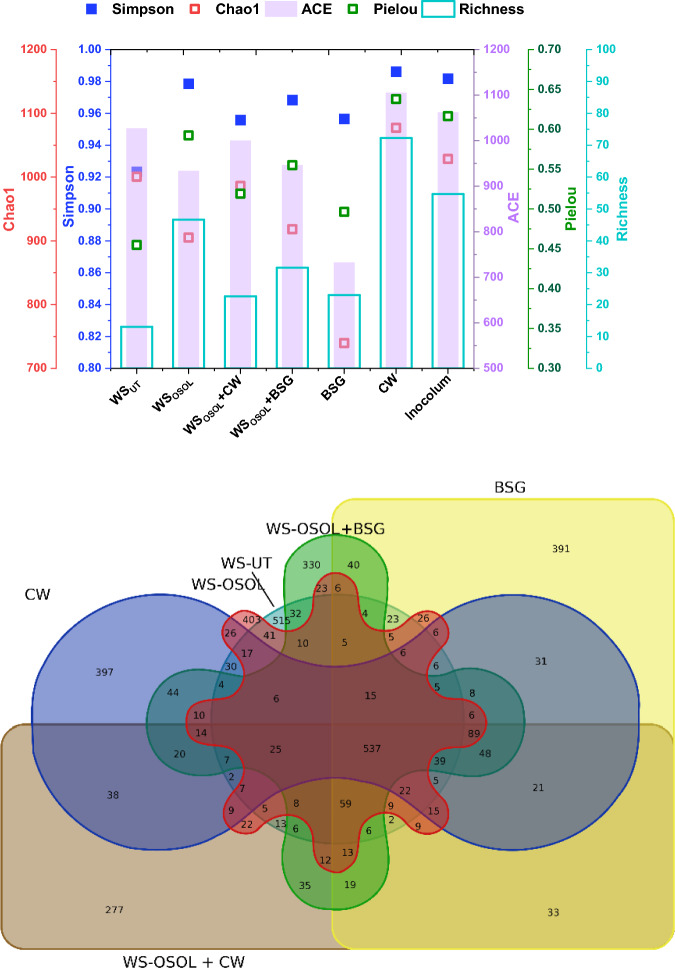
(**a**) Alpha diversity indices of reactors digesting three different substrates in mono and co-digestion mode in comparison with inoculum (**b**) Venn diagram illustrated the plot distribution patterns of OTUs that were shared among the six reactors.

In order to investigate how combining different types of cellulosic and protein-rich materials affects the structure of microbial communities, we conducted 16S rRNA gene amplicon sequencing. We compared the microbial profiles of the bacterial and archaeal communities in all six reactors. A Venn diagram depicted the distribution of operational taxonomic units (OTUs) and showed that 537 OTUs were present in all six reactors (Fig. 6). The reactor that contained organosolv-pretreated wheat straw (WS) and co-digested with cheese whey (CW) and brewer's spent grain (BSG) shared 403, 227, and 330 OTUs respectively. The number of unique OTUs in each reactor were as follows: 515 for WS only, 403 for WS with organosolv pretreatment (WS_OSOL_), 397 for CW, 391 for BSG, 330 for WS_OSOL_ combined with BSG, and 277 for WS_OSOL_ combined with CW. This unique OTU distribution influenced the degradation of substrates, as well as the production of biomethane and ammonium. The nature and chemical composition of the substrates led to diverse microbial compositions and interactions within the reactors^44^. A symbiotic relationship exists between acidogens and methanogens, as the volatile fatty acids and hydrogen/CO_2_ produced during acidogenesis/acetogenesis serve as precursor materials (acetate and hydrogen) for methane production. Moreover, acetogens have the capability to convert products from the acidogenic phase into acetic acid, CO_2_, and H_2_^11,45^. Additionally, the presence of amino acids in protein-containing substrates and their metabolism contribute to the enrichment of specific bacterial groups due to their synergistic impact during degradation.

**Figure 7:**
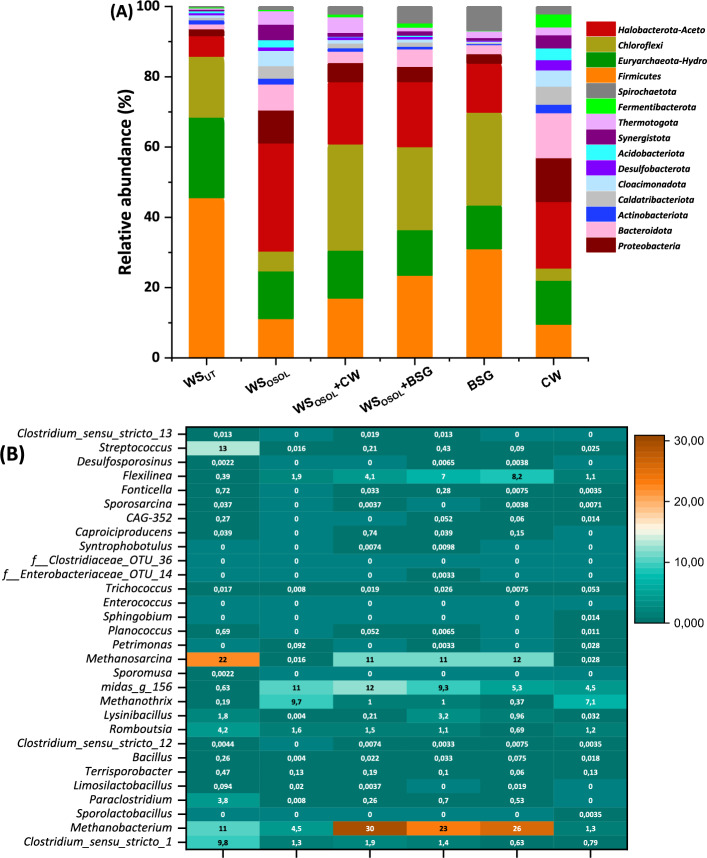
(**a**) Microbial community structure in six different reactors digesting wheat straw along with BSG and CW in mono and co-digestion mode, (**b**) the relative abundance of microbial heatmap for genera level.

After 24 days, significant changes were observed in the community structures of the reactors, indicating the influence of substrate type on the process and metabolites. Bacteria and archaea were the most prevalent at the phylum level in all the reactors; however, they were in different proportions. Based on taxonomic classification, the dominant bacterial phyla identified were *Firmicutes, Chloroflexi, Proteobacteria, Bacteroidota, Actinobacteriota, Caldatribacteriota, Cloacimonadota, Desulfobacterota, Acidobacteriota, Synergistota, Thermotogota,* and *Fermentibacterota*.Firmicutes alone accounted for 45.54% in the reactor fermenting CW, followed by 31.02% (WS_OSOL_ + CW), 23.41% (BSG), 16.97% (WS_OSOL_ + BSG), 10.97% (WS_OSOL_), and 9.26% (WS_UT_) of total effective sequences (Fig. 7). This trend indicates that reactors processing readily fermentable sugars over lignocellulosic biomass were dominated by Firmicutes. The archaea phylum consisted of *Euryarchaeota* and *Halobacterota*, with a dominance of 43.83% in the reactor loaded with WS_OSOL_ + BSG, followed by 40.23% with CW, 38.81% (WS_OSOL_ + CW), 36.47% (BSG), 18.8% (WS_OSOL_), and 15.52% (WS_UT_). The relative abundance of a particular phylum depended on the substrate being fed into the reactor, and the microbial community composition underwent a major shift by day 24.

**Figure 8:**
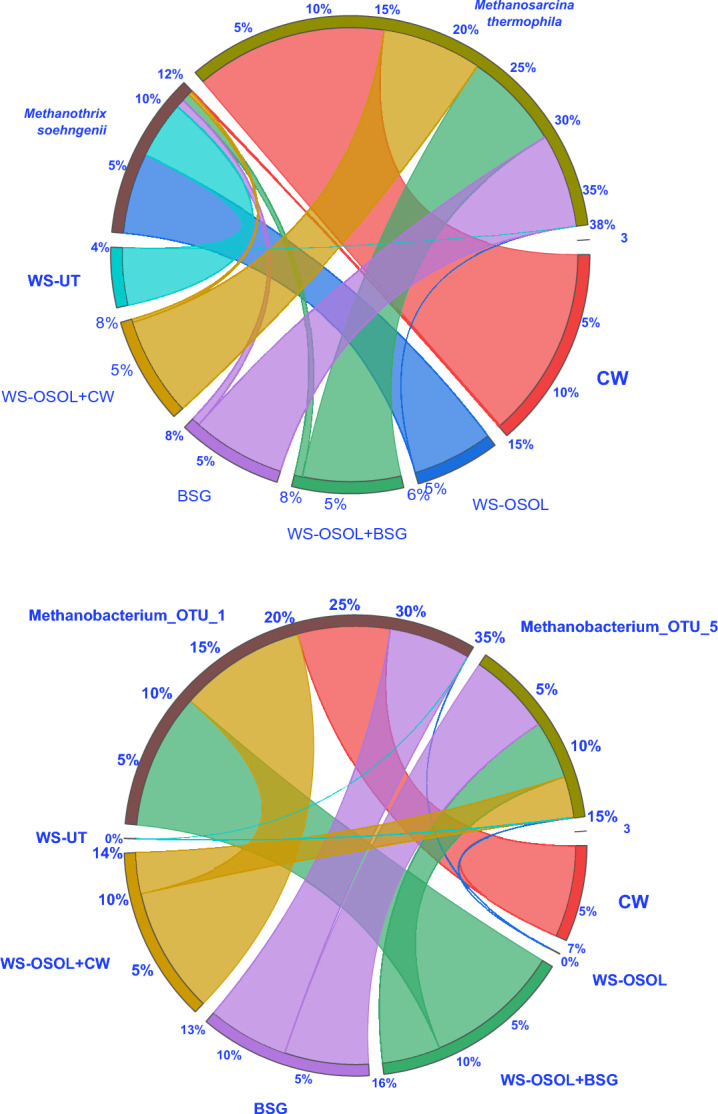
Chord diagram depicts the dominance of aceticlastic and hydrogenotrophic methanogens in the reactors loaded with different substrates operated in a mono and co-digestion mode for biomethane production.

Figure 7 shows the change in microbial communities at the genus level. The reactors that produced more methane had a high presence of Firmicutes compared to other bacterial groups. Firmicutes, particularly the Clostridiales, are well-known for their ability to ferment and break down substrates, producing monomers and organic acids that are needed for methane production. The dominance of Firmicutes (> 15%) was specifically seen in the reactors fed with protein-rich substances, which promoted the growth of Firmicutes. The microbial population in the reactor changed significantly with the variation in the concentration of acidogenic metabolites, indicating a higher abundance of Clostridia with volatile fatty acid (VFA) accumulation (CW: 25.5% > WS_OSOL_ + CW: 21.0% > BSG: 14.4% > WS_OSOL_ + BSG: 11.5% > WS_OSOL_: 9.4% > WS_UT_: 7.7%). Clostridia can efficiently produce VFAs from various organic materials such as food waste, lignocellulosic biomass, sucrose, starch, hemicellulose, glucose, cellulose, and sewage sludge^18,46^. The anaerobic digesters processing CW, co-digesting WS_OSOL_ + CW and WS_OSOL_ + BSG consistently demonstrate a methane yield surpassing 300 mL/gVS. This significant methane production can be directly attributed to the dominance of *Methanobacteriales, Methanosarciniales,* and *Methanomicrobiales*, all of which belong to the phyla *Euryarchaeota* (Fig. 8). These orders undeniably play a pivotal role in anaerobic digesters and are extensively documented to utilize CO_2_, H_2_, and formate for methane production^29,47,48^ (Eq. 1 and 2)

1 $${\text{CH}}_{{3}} {\text{COOH}} \to {\text{CH}}_{{4}} + {\text{ CO}}_{{2}}$$

2 $${\text{CO}}_{{2}} + {\text{H}}_{{2}} \to {\text{CH}}_{{4}} + {\text{ 2H}}_{{2}} {\text{O}}$$

The proliferation of *Methanosarcina*, an aceticlastic methanogen from the *Methanosarcinaceae* family, in the reactor demonstrated that the concentrations of ammonia and volatile fatty acids were well within the acceptable range. It is important to maintain these levels, as *Methanosarcina* is highly sensitive to these compounds^47^. The presence of high concentrations of ammonia can inhibit the aceticlastic pathway and promote the hydrogenotrophic pathway in specific microbial communities^49^. Aceticlastic methanogenesis, involving the direct conversion of acetate to methane and carbon dioxide by aceticlastic methanogens. Hydrogenotrophic methanogens use hydrogen and carbon dioxide as substrates for methane production. Aceticlastic methanogens can be negatively affected by high concentrations of ammonia (NH_4_^+^), which is toxic to them and interferes with their ability to use acetate for methane production^50^. In contrast, hydrogenotrophic methanogens demonstrate a higher tolerance to elevated ammonia levels, possibly due to reduced susceptibility to ammonia inhibition. The hydrogenotrophic methanogens, which are more tolerant to ammonia, can then utilize these fermentation products (hydrogen and carbon dioxide), leading to an increase in hydrogenotrophic methanogenesis.

Previous studies showed *Methanosarcina* as the predominant methanogens in wastewater containing high levels of acetate and ammonia, as well as in lipids-enriched wastewater^51,52^. Furthermore, the presence of *Methanobacterium*, a hydrogenotrophic microorganism from the *Methanobacteriaceae* family, clearly indicated the consumption of hydrogen in the reactor. Additionally, *Methanobacterium* has the capability to utilize alcohols as an electron donor and formate as an energy source. The presence of *Methanosarcina* from the *Halobacterota* phylum indicated the rapid consumption of volatile fatty acids for biomethane production.

The dominance of the species *Methanothrix soehngenii* and *Methanosarcina thermophila* was specifically observed in the following reactors: CW (22.61%), WS_OSOL_ + BSG (12.45%), WS_OSOL_ + CW (12.02%), and BSG (11.93%). This was well correlated with the acetate production and consumption rate in the total volatile fatty acids (VFAs) compared to the other reactors, with WS_OSOL_ at 9.49% and WS_UT_ at 6.73%. *M. soehngenii*, an aceticlastic methanogen, is known to be the most tolerant to acidic pH levels of 3.5 and 4.5^53^. However, in this study, the pH dropped to 5.1 from its initial pH of 7.5 during VFAs production, and further changed to 6.87 during the consumption of VFAs. *M. thermophila* is known for its versatility, being capable of growing on a variety of substrates including acetate, methanol, and methylamine. It can be found in a variety of feedstocks including municipal waste, wastewater, and sewage sludge^54^. The consumption of propionic and butyric acids in the reactors can be associated with the presence of *Synergistota*. By consuming these molecules, *Synergistota* help to keep the digestion process stable^55^. In summary, the synergy of substrates during the co-digestion of organosolv pretreated wheat straw with cheese whey and brewery spent grains significantly enhanced the relative abundance of dominant hydrolytic and acidogenic bacteria, followed by methanogenic archaea, thus producing more biogas and bioammonium in the digestate.

## Conclusion

The study presented an effective method for improving biogas production through co-digestion. Furthermore, this study demonstrates the significance of organosolv pretreatment in facilitating the efficient fractionization wheat straw, delivering readily available sugars for fermentation process. Co-digestion significantly enhanced the biomethane yield by increasing abundance of the dominant carbohydrate-hydrolytic bacteria in the mixed culture. Protein rich brewery spent grains co-digested with cellulosic rich organosolv pretreated wheat straw favored ammonium production in the digestate. Co-digestion facilitated abundance of aceticlastic methanogens in the reactor dosed with protein rich substrate assisting an enhanced transformation of volatile fatty acids to biomethane whereas hydrogenotrophs were enriched in the reactors employed with cellulosic rich substrate supporting conversion of CO_2_ and H_2_ to biomethane. Co-digestion strategy combining various substrate differed in their chemical nature was beneficial to regulate the process towards an enhanced microbial metabolites.

## Data Availability

All the data and materials generated during the present study are accessible upon request from the corresponding authors.
